# Affective Disorders, Psychosis and Dementia in a Community Sample of Older Men with and without Parkinson’s Disease

**DOI:** 10.1371/journal.pone.0163781

**Published:** 2016-09-30

**Authors:** Osvaldo P. Almeida, Kieran McCaul, Graeme J. Hankey, Bu B. Yeap, Jonathan Golledge, Leon Flicker

**Affiliations:** 1 School of Psychiatry & Clinical Neurosciences, University of Western Australia, Perth, Australia; 2 WA Centre for Health & Ageing of Centre for Medical Research, Harry Perkins Institute of Medical Research, Perth, Australia; 3 Department of Psychiatry, Royal Perth Hospital, Perth, Australia; 4 School of Medicine and Pharmacology, University of Western Australia, Perth, Australia; 5 Department of Neurology, Sir Charles Gairdner Hospital, Perth, Australia; 6 Department of Endocrinology, Fiona Stanley Hospital, Perth, Australia; 7 Queensland Research Centre for Peripheral Vascular Disease, College of Medicine and Dentistry, James Cook University, Townsville, Australia; 8 Department of Vascular and Endovascular Surgery, The Townsville Hospital, Townsville, Australia; 9 Department of Geriatric Medicine, Royal Perth Hospital, Perth, Australia; Oslo Universitetssykehus, NORWAY

## Abstract

**Background:**

Dementia and affective and psychotic symptoms are commonly associated with Parkinson’s disease, but information about their prevalence and incidence in community representative samples remains sparse.

**Methods:**

We recruited a community-representative sample 38173 older men aged 65–85 years in 1996 and used data linkage to ascertain the presence of PD, affective disorders, psychotic disorders and dementia. Diagnoses followed the International Classification of Disease coding system. Age was recorded in years. Follow up data were available until December 2011.

**Results:**

The mean age of participants was 72.5 years and 333 men (0.9%) had PD at study entry. Affective and psychotic disorders and dementia were more frequent in men with than without PD (respective odds ratios: 6.3 [95%CI = 4.7, 8.4]; 14.2 [95%CI = 8.4, 24.0] and 18.2 [95%CI = 13.4, 24.6]). Incidence rate ratios of affective and psychotic disorders were higher among men with than without PD, although ratios decreased with increasing age. The age-adjusted hazard ratio (HR) of an affective episode associated with PD was 5.0 (95%CI = 4.2, 5.9). PD was associated with an age-adjusted HR of 8.6 (95%CI = 6.1, 12.0) for psychotic disorders and 6.1 (95%CI = 5.5, 6.8) for dementia. PD and dementia increased the HR of depressive and psychotic disorders.

**Conclusions:**

PD increases the risk of affective and psychotic disorders, as well as dementia, among community dwelling older men. The risk of a recorded diagnosis of affective and psychotic disorders decreases with increasing age.

## Introduction

Parkinson’s disease (PD) is a neurodegenerative disorder that affects about 1% of the population aged 65 years or older [1]. The typical diagnostic features of PD include resting tremor, bradykinesia, rigidity, asymmetric onset of symptoms and postural instability [2], However, behavioural and psychological disturbances are common in PD and represent a major source of distress to patients and carers [3]. These include depressive or manic symptoms, psychosis and cognitive impairment.

Depressive symptoms affect 1 in 3 people with PD [4], although the prevalence of depressive disorders may be lower [5]. There is also uncertainty about the incidence rates of depression and other affective symptoms in this population because of the limited number, small sample sizes and short follow up of existing longitudinal studies [4].

Psychotic symptoms in the form of delusions and hallucinations are also commonly associated with PD. Clinic-based studies suggest that the lifetime prevalence of visual hallucinations among people with PD is as high as 50% [6], while the point-prevalence of delusions may range from 5 to 10% [7]. Again, only limited longitudinal data on the incidence of psychotic symptoms from community representative samples of people with PD are available [8].

Dementia is another common neuropsychiatric complication of PD that affects 25–30% of patients in cross-sectional studies [9]. Preliminary evidence suggests that nearly half of those with PD develop dementia within 10 years, and that only 15–20% of patients remain free of overt cognitive impairment after 20 years [10]. However, few longitudinal studies have been population-based [11,12], and none has stratified the rates of dementia by age. As the risk of dementia increases with increasing age [13], it is important to clarify if PD interacts with age to modulate the risk of dementia later in life.

Furthermore, if the neurodegenerative processes associated with dementia in PD lie in the causal pathway that leads to the expression of depression, mania and psychosis, then these symptoms should become more prevalent in people with PD who develop dementia. In other words, PD and dementia (which could be viewed a surrogate clinical marker of the extent of neurodegeneration) would be expected to have either additive or multiplicative effects on the incidence of affective and psychotic disorders associated with PD.

We undertook an electronic data-linkage study of a community sample of nearly 40000 older people to clarify the prevalence and incidence of dementia and of affective and psychotic symptoms among men with and without PD. We hypothesised that the incidence of the recorded diagnosis of dementia, affective and psychotic disorders would increase with increasing age (because the prevalence of PD increases with age), and that dementia would interact with PD to enhance the risk of these conditions.

## Methods

### Study design and setting

This cohort study recruited participants between April 1996 and November 1998, and followed them until an event of interest, death or the 1^st^ December 2011, whichever occurred first. All participants were community dwelling and, at the time of enrolment, were living in the Perth metropolitan region of Western Australia.

### Participants

We used the Australian Electoral roll to produce a list of all men aged 65–85 years living the Perth metropolitan region in 1996 (registration to vote is compulsory for all Australian citizens aged 18 years or older). Women were not included in the sample because the primary aim of the original study was to investigate the impact of screening on the outcome of abdominal aortic aneurysm (which is much more frequent in men than women). Of the 49801 potential participants, 1839 had died by the time the study started and another 9482 were not selected because they were living outside the immediate Perth metropolitan region. Of the remaining 38480 men, 307 were excluded because they were younger than 65 years (these men were invited, in error, before completing their 65^th^ birthday), leaving a total study sample of 38173 older men. Further details about the study design and characteristics of the sample have been reported elsewhere [14]. The study was approved by the Ethics Committees of the University of Western Australia and of the Department of Health of Western Australia, as well as by the Legal Data Custodian of Western Australia. As participants were de-identified and followed using administrative data, no written consent was obtained. The Ethics Committees of the University of Western Australia and of the Department of Health of Western Australia approved the study procedures. The Legal Data Custodian of Western Australia is responsible for ensuring that study data are de-identified and used solely for the purposes of approved medical research. In addition, the Legal Data Custodian is responsible for ensuring that named investigators only use the data.

### Study measures

We used the Western Australian Data Linkage System (WADLS) to retrieve data about the diagnosis of PD, as well as occasions of service associated with the diagnoses of dementia and affective and psychotic disorders. WADLS brings together data for all occasions of service received by Western Australians (inpatient and outpatient mental health services, hospital morbidity data and the death registry) [15]. WADLS does not include socio-demographic or lifestyle information due to privacy safeguards, only health events. The coding of clinical diagnoses and procedures in WADLS was recorded according to the International Classification of Diseases (ICD) 8^th^ edition from 1^st^ January 1966 to 31^st^ December 1969, the 9^th^ edition from 1^st^ January 1970 to 30^th^ June 1999, and the 10^th^ edition from the 1^st^ July 1999. Medical practitioners ascribe the medical diagnoses associated with all occasions of health service received by Western Australians, which are then coded and incorporated into WADLS.

For the purposes of this study, we created three outcome groups: affective disorder (depressive, bipolar or organic affective disorder), psychotic disorder (schizophrenia, schizoaffective, delusional and organic hallucinosis or delusional disorder) and dementia. These included the following diagnostic categories:

Affetive disorders:

- depressive disorder: ICD-8 codes 296.0 and 300.4; ICD-9 codes 296.2, 296.3, 300.4 and 311; ICD-10 codes F32, F33, F34.1, F38.10;
- bipolar disorder: ICD-8 codes 296.1 and 296.3; ICD-9 codes 296.0, 296.1, 296.4, 296.5, 296.7, 296.80 and 296.81; ICD-10 codes F30 and F31;
- organic affective disorder: ICD-9 codes 290.13, 290.21, 290.43, 292.84, 293.83; ICD-10 codes F00-F03.x3 and F06.3;

Psychotic disorders:

- schizophrenia, schizoaffective and delusional disorder: ICD-8 and ICD-9 codes 295 and 297; ICD-10 codes F20, F22, F23, F25, F28 and F29;
- organic hallucinosis or organic delusional disorder: ICD-8 codes 292–294; ICD-9 codes 290.12, 290.42, 292.1, 293.81, 293.82; ICD-10 codes F00-F03.x1 or 2, F06.0, F06.1, F06.2, F10-F19.5, F10-F19.7;

Dementia:

- dementia: ICD8 code 290; ICD-9 codes 290, 294.1, 294.2, 331.0, 331.1, 331.2, 331.82; ICD-10 codes F00-F03, G30, G31.0, G31.1, G31.83.

The diagnosis of Parkinson’s disease was the principal exposure of interest of the study, and its presence was established according to the following ICD codes: 342 (ICD-8), 332 (ICD-9) and G20 (ICD-10). We calculated the age of participants as the difference, in years, between the date of enrolment and the date of birth. We stratified age into 5-year blocks: 65–69, 70–74, 75–79, 80–84 and ≥85 years. We followed the same approach to determine the age, and age stratum, of men at the time of the diagnosis of PD.

### Statistical analyses

We used the statistical software Stata 14.1 (StataCorp LP, 2015) to manage and analyse the data. Descriptive statistics summarised categorical variables as count and proportions (%), and continuous variables as mean, range, and standard deviation of the mean (SD). We employed t-tests to compare the age of participants with and without PD, and reported the t-statistic, the number of degrees of freedom (df) and p-value. We used logistic regression to investigate the odds of dementia and affective and psychotic disorders in men with compared with those without PD, and Cox regression to investigate the hazard ratio (HR) of these outcomes over time. Incident cases of PD contributed data as controls until the diagnosis of PD was established, and were subsequently followed as cases until the event, death or the 1^st^ December 2011, whichever occurred first. We used the stsplit command of Stata for this purpose. In addition, we calculated the age-standardised incidence rate of dementia, affective and psychotic disorders per 1000 person-years for each age stratum, and again took into account new cases of PD arising during follow up. Incidence rate ratios were estimated from 2x2 tables. The analyses for incident affective and psychotic disorders and dementia excluded cases with recorded history of these conditions at study entry. We also used logistic regression to investigate whether depression occurred more frequently in the 2 years before the diagnosis of PD—the results are reported as odds ratio. All analyses were adjusted for the age of participants. We reported the 95%CI of all statistical tests and risk estimates.

## Results

The mean age of the 38173 participants was 72.5 years (SD = 4.6) and 333 (0.9%) of them had a recorded diagnosis of PD at study entry. Men with PD were, on average, 2.8 (95%CI = 2.3, 3.3) years older than men without PD (t = 11.30, df = 38171, p<0.001). Table 1 summarises the clinical characteristics of the study cohort and of men with PD. The latter had higher prevalence of affective and psychotic disorders, as well as of dementia.

**Table 1:** Clinical characteristics of the study cohort and of men with Parkinson’s disease at the start of the follow up period.

		Population	Parkinson’s disease	Odds Ratio
		N = 38173	N = 333	(95%CI)
		n (%)	n (%)	
Age group (years)	65–69	13389 (35.1)	51 (15.3)	1 (Reference)
	70–74	13116 (34.4)	95 (28.5)	1.9 (1.4, 2.7)
	75–79	8719 (22.8)	122 (36.6)	3.7 (2.7, 5.2)
	≥ 80	2949 (7.7)	65 (19.5)	5.9 (4.1, 8.5
Affective disorders		1270 (3.3)	59 (17.7)	6.3 (4.7, 8.4)*
Psychotic disorders		169 (0.4)	17 (5.1)	14.2 (8.4, 24.0)*
Dementia		405 (1.1)	63 (18.9)	18.2 (13.4, 24.6)*

*Odds ratio adjusted for age group.

**Fig 1:**
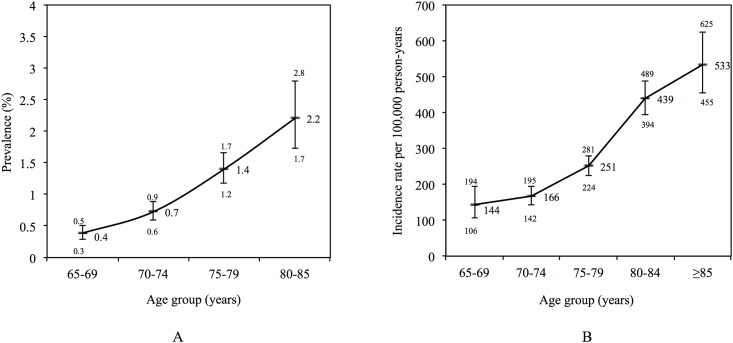
Panel A: the diamonds depict the percentage prevalence of Parkinson’s disease for each age group and the whiskers the 95% confidence interval of the prevalence estimate. Panel B: the diamonds depict the incidence rate of Parkinson’s disease by age group per 100,000 person-years, and the whiskers the 95% confidence interval of the rates.

**Fig 2:**
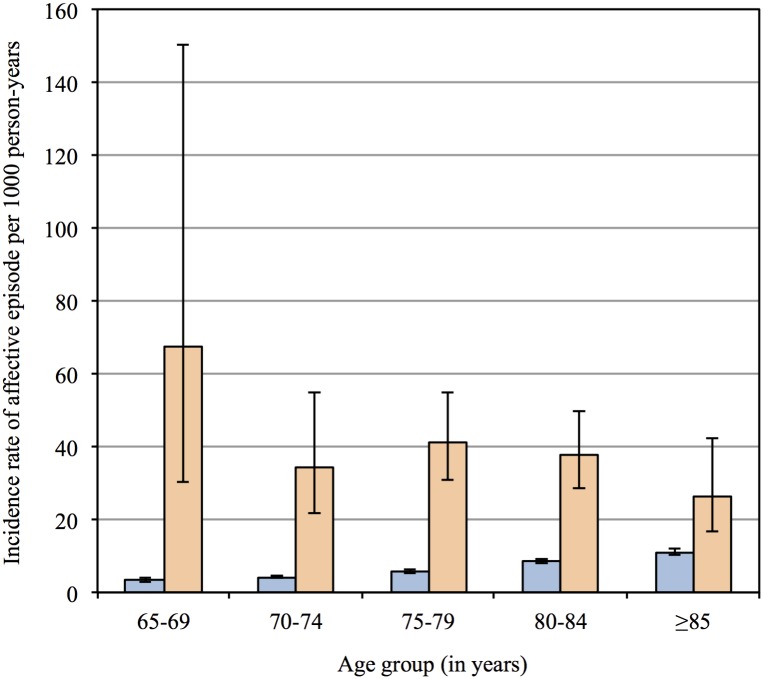
The bars depict the incidence rate of affective episodes per 1000 person-years for each age group, and the whiskers represent the 95% confidence interval of the rates. The left and right bars for each age group show the results for men without and with Parkinson’s disease respectively.

In addition to the 333 prevalent cases of PD at study entry, another 1119 received the diagnosis of PD during the follow up period. Fig 1 provides detailed information about the prevalence and incidence of PD by age group. Two thousand five hundred and fifty-eight men (6.9%) experienced an incident affective episode, with an excess of episodes affecting participants with PD (11.9%). The age-adjusted incidence rates per 1000 person-years of an affective episode were 36.9 (95%CI = 31.2, 43.6; total = 3688 person-years) and 6.5 (95%CI = 6.2, 6.7; total = 374239 person-years) for participants with and without PD, and the incidence rate ratio of affective disorder associated with PD was 5.7 (95%CI = 4.8, 6.8) relative to men without PD. Fig 2 shows the age-adjusted incidence rates of an affective episode by age group. Of the 2558 new episodes of affective disorder recorded during follow up, 2477 were due to depression, while among men with PD the respective figures were 255 and 246 (i.e., only 9 of the recorded affective episodes were ascribed to bipolar disorder). The incidence rate ratios of affective disorder among men with PD relative to men without PD declined with increasing age from 20.4 (95%CI = 7.3, 46.0) to 8.1 (95%CI = 4.7, 13.0), 7.2 (95%CI = 5.2, 9.7), 4.4 (95%CI = 3.2, 5.9) and 2.4 (95%CI = 1.4, 3.9) for participants aged 65–69, 70–74, 75–79, 80–84 and ≥85 years. The overall age-adjusted HR of an affective episode among men with PD was 5.0 (95%CI = 4.2, 5.9) relative to men without PD.

**Fig 3:**
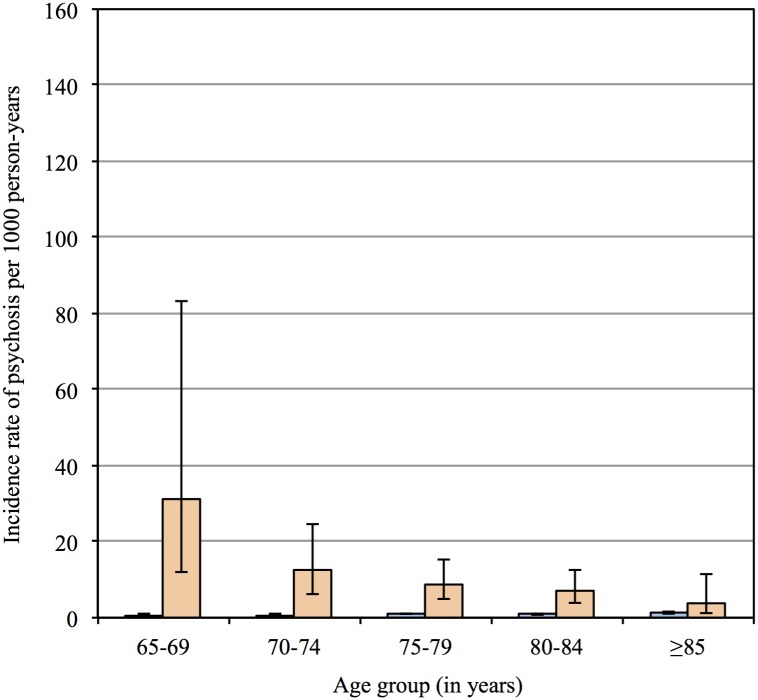
The bars depict the incidence rate of psychotic episode per 1000 person-years for each age group, and the whiskers represent the 95% confidence interval of the rates. The left and right bars for each age group show the results for men without and with Parkinson’s disease respectively.

Likewise, 402 participants had a new-recorded diagnosis of psychosis during follow up, of whom 58 had PD. The age-adjusted incidence rate of psychosis per 1000 person-years was 8.4 (95%CI = 6.1, 11.5; total = 4522 person-years) and 0.9 (95%CI = 0.8, 1.0; total = 388528 person-years) for men with and without PD, and the incidence rate ratio was 9.0 (95%CI = 6.2, 12.5). Fig 3 shows the age-adjusted incidence rates of psychosis for men with and without PD by age group. The incidence rate ratios of psychosis for men with PD relative to men without were 62.0 (95%CI = 15.0, 194.5), 16.6 (95%CI = 6.9. 34.5), 8.3 (95%CI = 4.2, 15.0), 7.6 (95%CI = 3.7, 14.3) and 2.8 (95%CI = 0.6, 8.5) for participants aged 65–69, 70–74, 75–79, 80–84 and ≥85 years. The overall age-adjusted HR of psychosis associated with PD was 8.6 (95%CI = 6.1, 12.0).

**Fig 4:**
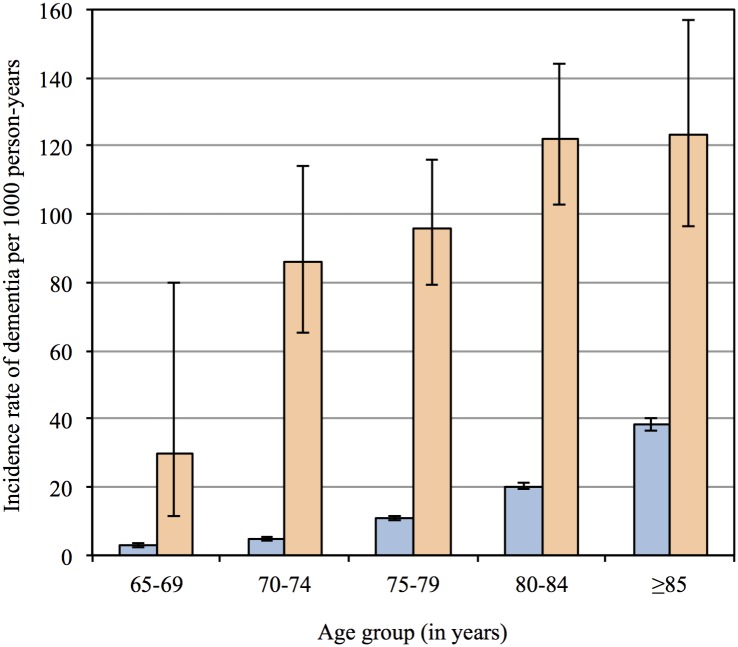
The bars depict the incidence rate of dementia per 1000 person-years for each age group, and the whiskers represent the 95% confidence interval of the rates. The left and right bars for each age group show the results for men without and with Parkinson’s disease respectively.

A total of 5678 men developed dementia during follow up (15.0%), of whom 357 had PD. The age-adjusted incidence rate of dementia was 104.2 (95%CI = 93.9, 115.6; total = 3426 person-years) and 14.0 (95%CI = 13.6, 14.4; total = 379848 person-years) per 1000 person-years for men with and without PD, and the incidence rate ratio was 7.4 (95%CI = 6.7, 8.3). Fig 4 depicts the age-adjusted incidence rates of dementia by age group for participants with and without PD. The incidence rate ratios of dementia for men with PD relative to men without were 10.3 (95%CI = 2.7, 27.3), 18.2 (95%CI = 13.2, 24.4), 8.9 (95%CI = 7.2, 10.8), 6.0 (95%CI = 5.0, 7.1) and 3.2 (95%CI = 2.5, 4.1) for participants aged 65–69, 70–74, 75–79, 80–84 and ≥85 years. The overall age-adjusted HR of dementia associated with the diagnosis of PD was 6.1 (95%CI = 5.5, 6.8).

PD and dementia had significant independent effects on the onset of affective disorders (HR = 5.9 [95%CI = 4.6, 7.6] and 3.4, [95%CI = 3.1, 3.7], respectively), although the interaction term indicated that men with PD who also had dementia were less likely to receive the diagnosis of depression (HR = 0.4, 95%CI = 0.3, 0.5). Similarly, our analyses showed that PD and dementia were independent risk factors for incident psychotic symptoms (HR = 15.0 [95%CI = 8.8, 25.6] and 9.1 [95%CI = 7.4, 11.3]), but men with PD who also had dementia had lower probability of receiving the diagnosis of a psychotic syndrome (HR = 0.2, 95%CI = 0.1, 0.3). All analyses were adjusted for age.

### Duration of PD and mental health

We completed a series of additional analyses to investigate the association between recency of the diagnosis of PD and the onset of mental health symptoms. Fourteen men had an affective episode recorded within 1 year of the diagnosis of PD (rate = 178/1000 person-years compared with 6/1000 person-years for men without PD), with PD being associated with an age-adjusted HR of an affective episode of 22.7 (95%CI = 13.4, 38.4) relative to men without PD. In contrast, the rate of depression among men with history of PD > 1 year was 34/1000 person-years (n = 122), and the age-adjusted HR of an affective episode compared with men without PD was 4.6 (95%CI = 3.8, 5.5). As previous reports have suggested that depression could precede the diagnosis of PD by about 2 years [16], we used logistic regression to investigate this possible association. The age-adjusted odds ratio of depression occurring in the 2 years preceding the diagnosis of PD was 1.03 (95%CI = 0.82, 1.29).

Similarly, the rate of psychosis within the first year of diagnosis of PD was greater in men with than without PD (41/1000 person-years vs 1/1000 person-years; n = 4 vs n = 358), with the diagnosis of PD being associated with 41.2 increase in the age-adjusted HR of psychosis (95%CI = 15.3, 110.7) within the first year following diagnosis. The rate of psychosis for men with history of PD > 1 year was 8/1000 person-years (n = 34), and the respective age-adjusted HR = 7.9 (95%CI = 5.6, 11.3) compared with men without PD.

The rate of dementia within the first year of diagnosis of PD was 344/1000 person-years (n = 22) compared with 14/1000 person-years for men without PD (n = 5150). The age-adjusted HR of dementia among men with PD ≤ 1 year compared with men without PD was 17.4 (95%CI = 11.4, 26.5). The age-adjusted HR of dementia after the 1^st^ year of diagnosis of PD was 6.0 (95%CI = 5.4, 6.7).

## Discussion

Our survey showed that mood and psychotic disorders, as well as dementia, were more prevalent in older men with than without PD. We also found that the incidence rates of mood disorders and psychosis associated with PD were highest in the young old, while the rates for dementia moved in the opposite direction. The incidence rates of depression, psychosis and dementia were 6 to 9 times higher in men with than without PD, although the discrepancy between the groups became less pronounced with increasing age. Contrary to our prediction, the interaction between PD and dementia was associated with reduced age-adjusted hazard of both affective and psychotic disorders. In addition, the risk of affective episodes, psychosis and dementia were highest during the first year of diagnosis of PD.

### Strengths and limitations

This study has the merit of having had access to the entire male population of older Australian citizens living in the Perth metropolitan region, although people living in aged care facilities and foreign nationals were not included. There are no reliable data about the number of people with PD living in residential care facilities in Australia, but indirect estimates suggest a figure around 7–8% [17]. In addition, there is no evidence that the health outcomes of older foreign nationals (approximately 20% of this age-group) are different from their Australian peers [18]. This, together with the exclusion of men younger than 65 years and the possibly low sensitivity of WADLS to detect PD in the early stages of illness (the system does not collect primary care data), may raise concerns that our approach to establish the presence of PD may have been suboptimal. Nonetheless, both the prevalence and incidence of PD in this cohort are consistent with the those of European and American surveys [1]. It is also difficult to imagine that a man with PD would have been admitted to residential care without having had a prior contact with the health services, which suggests that our approach to case ascertainment was acceptable (albeit not ideal). A notable strength of our study design is that longitudinal information was available for all participants through WADLS, as the internal and external migratory movement of older Western Australians is negligible [19].

WADLS yields valid mental health diagnoses, particularly for severe mental disorders [20]. Nonetheless, its sensitivity and specificity to detect behavioural and psychological symptoms associated with PD has not been documented. Furthermore, the coding of clinical syndromes is potentially open to some idiosyncrasies. For example, a major depressive episode associated with PD could have been recorded as F32, F33 (recurrent depressive disorder) or F06.3 (organic affective disorder, which does not have a mood specified: depression or mania). For this reason, we grouped all affective episodes into one single category (affective disorders), even though most seem to have been due to depression. Similar issues apply to the coding of psychotic states, which were grouped under the syndromic category of ‘psychotic disorders’. We also refrained from looking at specific causes of dementia in this study because the unspecified code for dementia (F03 or 290) was used frequently (data not shown). Of relevance to these analyses is the potential association between PD and dementia with Lewy bodies. None of our participants received the diagnosis of dementia with Lewy bodies alone, which suggests that these cases were most likely included in the unspecified dementia group. Finally, we did not attempt to investigate the association between PD and anxiety disorders because these cases are only infrequently managed within the hospital system of Western Australia, hence the sensitivity of WADLS for these cases would most likely have been unacceptably low.

We must also consider the possibility of Berkson’s bias [21]. For example, health professionals who manage older people with PD may be more attuned than other health specialists to the assessment of mental state (i.e., higher sensitivity for case detection in people with than without PD), particularly during the initial period of diagnostic workup and management. This could lead to an inflated risk ratio of mental disorders associated with PD. The risk of receiving a mental health diagnosis associated with PD was highest during the first year of illness, and this could indicate the presence of Berkson’s bias or represent an early response to diagnosis or treatment. Of note, the rates of dementia diagnosed during the first year of PD did not increase with increasing age, but rates increased progressively with increasing age for men without PD and for those who had had a diagnosis of PD for more than 1 year (data not shown). Taken together, these findings suggest that some detection bias may have occurred, particularly during the first year following the diagnosis of PD. However, the magnitude of the associations that we observed and the consistency of our results with those of others [6,9,22] suggest that this type of bias alone would be insufficient to explain the higher incidence of mental disorders among men with PD.

Finally, we acknowledge that this survey was limited to older men, so that we cannot be certain that our findings would apply equally to women or younger men with PD.

### Interpretation

More men with than without PD experience affective and psychotic symptoms and cognitive impairment in later life. The cross-sectional and longitudinal association between PD and these neuropsychiatric outcomes suggest that this link might be causal. As the prevalence and incidence of PD increases with increasing age [1], one would expect a similar rise in the incidence of affective and psychotic disorders with increasing age. That was not the case. These results could be interpreted as indicating that the neurodegenerative, physiological and environmental factors associated with PD play a lesser role in the causation of neuropsychiatric symptoms as people age, possibly because of the increased contribution of other risk factors associated with getting older and with disease progression (e.g., comorbidity, polypharmacy or use of dopaminergic agonists) [23,24]. Differential mortality, or survivorship bias, could be another possible explanation for our findings. People with comorbid PD and depression/psychosis may have greater mortality than their counterparts without those symptoms [25,26], so that an increasing number of PD survivors may have uncomplicated illness at advanced ages due to the early censoring of those with psychosis and depression. It is also possible that affective and psychotic disorders are viewed as an integral part of the presentation of people with longstanding PD or dementia, so that no specific coding is recorded for these disorders, making them less frequent as the severity of PD increases (a form of ascertainment bias). Hence, our findings show the prevalence and incidence of the recorded psychiatric diagnoses received by older men with and without PD or dementia—they may not necessarily reflect the true prevalence of the affective and psychotic syndromes in this population.

The results of our study have also shown that the risk of dementia is greatly increased in men with PD. Others had already shown that about ¾ of older people with PD develop dementia over an 8-year period [27], with data from the Sydney Multicentre Study indicating that conversion to dementia occurs more quickly amongst those with late onset PD [28], although the increased incidence of dementia in very late life may not be due to the presence of PD alone. A recent review of the pathological changes associated with dementia in PD concluded that alpha-synuclein pathology interacts with tau and amyloid changes to modulate dementia risk [29]. Indeed, about 1/3 of those with dementia associated with PD fulfill pathological criteria for Alzheimer’s disease [30]. Nonetheless, the high rate of dementia found during the first year following the diagnosis of PD suggests that some of these men may have had dementia with Lewy bodies.

In summary, PD is associated with increased risk of affective and psychotic disorders, as well as of dementia. Both PD and dementia increase the risk of affective and psychotic disorders in later life. Regardless of the age of onset of PD, our results suggest that clinicians should investigate the possible presence of neuropsychiatric symptoms throughout the course of the illness. Dementia is also a common, but not inevitable, occurrence in older men with PD, with the presence of PD becoming less relevant in determining the onset of dementia as the population ages. These findings suggest that changes associated with older age play an increasingly prominent role in modulating dementia risk later in life, so that factors that decrease the risk of dementia in the general population may also contribute to decrease the risk of dementia in people with PD.
